# Follicular reconstruction and neo-oogenesis in xenotransplantation of human ovarian isolated cells derived from chemotherapy-induced POF patients

**DOI:** 10.1186/s13036-023-00384-2

**Published:** 2023-11-20

**Authors:** Sara Khaleghi, Farideh Eivazkhani, Somayeh Tavana, Ashraf Moini, Marefat Ghaffari Novin, Petkov Stoyan, Hamid Nazarian, Rouhollah Fathi

**Affiliations:** 1 Department of Biology and Anatomical Sciences, School of Medicine, Shahid Beheshti University of Medical Sciences, Tehran, Iran; 2 Department of Embryology, Reproductive Biomedicine Research Center, Royan Institute for Reproductive Biomedicine, ACECR, Tehran, Iran; 3 Department of Endocrinology and Female Infertility, Royan Institute of Reproductive Biomedicine, ACECR, Tehran, Iran; 4 Breast Disease Research Center (BDRC), Tehran University of Medical Science, Tehran, Iran; 5 Platform Degenerative Diseases, German Primate Center, GmbH, Leibniz Institute for Primate Research, Göttingen, 37077 Germany; 6 German Center for Cardiovascular Research (DZHK), Partner Site Göttingen, Göttingen, 37077 Germany

**Keywords:** Xenotransplantation, Ovarian follicle, Oogenesis, Chemotherapy, Oogonial stem cells

## Abstract

**Background:**

Developing new strategies to restore fertility in patients with chemotherapy-induced Premature Ovarian Failure (Chemo-POF) is important. We aimed to construct an Artificial Ovary (AO) by seeding Human Ovarian Cortical Cells (HOCCs) into Human ovarian Decellularized Cortical Tissue (DCT). We assessed the AO’s ability to produce new ovarian follicles following xenotransplantation to NMRI mice.

**Material and methods:**

The DCTs were prepared, and cell removal was confirmed through DNA content, MTT assay, DAPI and H&E staining. Next, HOCCs were isolated from both Chemo-POF and Trans (as a control group) ovarian patients. The HOCCs were characterized using immunostaining (FRAGILIS, Vimentin, and Inhibin α) and real time PCR (*DDX4*, *STELLA*, *FRAGILIS, Vimentin, FSH-R, KI67)* assays. The HOCCs were then seeded into the DCTs and cultured for one week to construct an AO, which was subsequently xenotransplanted into the mice**.** The existence of active follicles within the AO was studied with H&E and immunofluorescence (GDF9) staining, Real-time PCR (*GDF9*, *ZP3*) and hormone analysis (Estradiol, FSH and AMH).

**Results:**

The results of gene expression and immunostaining showed that 85–86% of the isolated cells from both Trans and Chemo-POF groups were positive for vimentin, while 2–5% were granulosa cells and OSCs were less than 3%. After xenotransplantation, histological study confirmed the presence of morphologically healthy reconstructed human ovarian follicles. Additionally, immunofluorescence staining of GDF9 and hormonal assay confirmed the presence of secretory-active follicles on the AO.

**Conclusion:**

Our findings demonstrate that an artificial ovary produced by seeding HOCCs on DCT can support HOCCs proliferation as well as neo-oogenesis, and enable sex hormone secretion following xenotransplantation.

## Introduction

Premature Ovarian Failure (POF) is a condition that impedes ovarian function before the age of 40 and leads to infertility in women of child-bearing age. The POF patient's ovaries stop producing eggs and hormones, and such women experience amenorrhea, hypoestrogenism and elevated gonadotropin levels [1–3]. POF can be spontaneous or induced by iatrogenic reasons such as chemotherapy or radiotherapy treatments for cancer [1, 4].

Generally, 1 in 6 women develop cancer during their lifespan, and the disease is estimated to affect 4.38 million people worldwide [5]. It has been recognized that cancer therapy provided to women younger than 45 years of age, may lead to distinct and permanent damages to the ovaries [6]. Chemotherapy-induced cytotoxicity can damage dividing cells and kill growing follicles in the ovaries. Chemotherapy may also induce inflammation and destruction of vascular and stromal cells of the ovaries. Furthermore, some studies have shown that the extracellular matrix (ECM) of the ovarian tissue undergoes a series of structural changes due to cancer and chemotherapy, which can damage the ovarian cells and follicles [7, 8].

Ovarian tissue cryopreservation before anticancer therapies and autotransplantation carries the risk of carrying malignant cells, which makes transplantation after disease remission inadvisable [9]. New strategies are required to avoid reimplanting malignant cells and restore fertility in these patients. The construction of an artificial ovary (AO) is an emerging strategy that may help achieve these goals.

The growing interest in ovarian tissue engineering as a field of research, which focuses on the development of artificial ovaries. Artificial ovaries are constructed, which involves coupling follicles- and/or isolated cells with implantable biomimetic scaffolds that can support follicle proliferation and ensure sex hormone secretion . The different types of scaffolds used in artificial ovary construction, including synthetic materials such as polyethylene glycol and polyvinyl alcohol, natural matrices such as agar, alginate, and hyaluronic acid, and tissue-derived ECM that support cell growth and folliculogenesis. The sentence goes on to mention that these different types of scaffolds are used to mimic the natural structure of the ovary [10–13]. Decellularized tissue-derived ECM has been considered a practical solution because the science is already known, and the matrix is cost-effective, biocompatible, biodegradable, and would result in negligible levels of unfavorable immune response due to the absence of cells. These advantages have led to the extensive study and use of decellularized ECMs in tissue engineering [14–16].

Decellularized ECM-based scaffolds, derived from tissues such as muscle, spleen, liver, and ovary have become promising tools in tissue engineering and regenerative medicine in recent years. The native biochemical and ultrastructural features of these tissues can be preserved in decellularized ECM-based scaffolds, which may contribute to their effectiveness as tools for tissue engineering and regenerative medicine [17–21]. The specialized role of ovarian ECM is providing both architectural support and a specialized microenvironment for regulating and controlling cellular activities and follicular development characteristics. Ovarian ECM plays in regulating follicular development, including morphology, survival, aggregation, proliferation, communication, and steroidogenesis [18, 22]. It has been reported to support a higher rate of follicular growth in both in vitro and in vivo conditions [17, 23, 24]. Additionally, human decellularized ovarian scaffold has been used as a natural microenvironment for various cellular processes, including attachment, penetration, expansion, and differentiation to tissue-specific cells. Alshaikh et al., demonstrated that ovarian scaffolds that support MSCs and stroma cells can support granulosa cells and estrogen production [25]. MacDonald et al., have been shown that culture of human oogonial stem cells (hOSCs) on a laminin-based ECM exhibited increased differentiation into in vitro derived oocytes [22].

Decellularized ovarian ECMs can be repopulated with various types of ovarian cells, including oogonial stem cells (OSC), stromal cells, and granulosa cells, to create a restructured organ or artificial ovary. Additionally, decellularized ovarian ECM provide a good 3D microstructure for regenerating ovarian follicular activity [18, 19, 26].

The aim of the current study is to develop a transplantable artificial ovary by combining human ovarian Decellularized Cortical Tissue (DCT) with seeded human ovarian cortical cells (HOCCs), and to investigate the ability of this artificial ovary to produce new ovarian follicles following xenotransplantation to NMRI mice.

## Materials and methods

### Ethics approval

The study received approval from the Research Ethical Committee of the Royan Institute, Tehran, Iran (IR.ACECR.ROYAN.REC.1398.038) and adhered to the ethical guidelines of the Helsinki Declaration. The current study has been reported in accordance with the ARRIVE guidelines. Furthermore, it was conducted only after obtaining informed and signed consent from patients for the use of ovarian samples in experimental research.

### Group design

#### Trans group (control)

HOCCs were isolated from transsexuals and seeded into DCT (trans HOCCs + DCT).

#### Chemo-POF group

HOCCs were isolated from chemotherapy-induced POF patients and seeded into DCT (Chemo-POF HOCCs + DCT).

A total of donated cortical tissues were acquired from 8 women with Chemo- POF and 15 transsexuals individuals, with a mean age of 31 years and a range of 22–40 years. Additionally, the diagnosis of premature ovarian failure is made when patients experience secondary amenorrhea, which is characterized by consistently elevated FSH levels exceeding 40 IU/L and low estradiol levels.

### Patients and samples

**Fig. 1:**
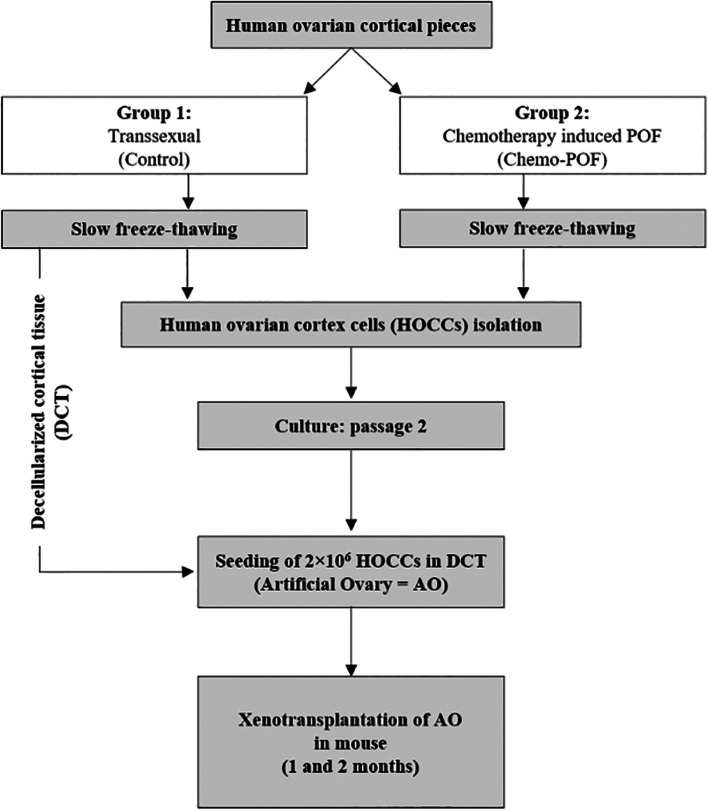
Experimental design

The experimental design of the study was shown in Fig. 1. Biopsies of donated human ovarian tissues were taken from two groups of patients: Chemo-POF women and transsexuals undergoing laparoscopic surgery for benign gynecologic disease. Tissues were transported from Arash hospital (Tehran, Iran) to the Ovarian Tissue Bank (OTB) of Royan Institute (Tehran, Iran) within an hour in DMEM/F12 + GlutaMAX (Dulbecco's Modified Eagle Medium, Gibco, Paisley, UK) on ice. The ovarian medullas were separated from the cortical part with a surgical scissor. The separated cortexes were cut into 5 × 5 × 5 mm strips. The strips were slow-frozen according to the standard protocol of OTB [27] until thawed before subsequent in vitro and in vivo experiments.

### Experimental animals

Twenty female NMRI mice (6–8 week) (NMRI; naval medical research institute) were purchased from the Royan Institute's animal house. The animals were fed pellets and water using an ad libitum method. The mice were kept under controlled 12- hour light/12-h dark cycles at 20–22˚C. The animals were anesthetized using xylazine/ketamine (100 mg/kg and 10 mg/kg, respectively) (Alfasan, Netherland; 960λ-640λ/kg), and after surgery, they were single housed for up to two months. All procedures were performed under animal care and ethics of Royan Institute.

### Decellularization of human ovarian cortical tissue

Ovarian cortical tissues (OCT) were collected from transsexual humans for decellularization. Initial and final wet weights were recorded for individual tissue pieces for DNA content normalization. The sizes of ovarian cortical pieces were ~ 5 × 5 × 5 mm. The thawing procedure was performed on cryopreserved human ovarian cortical stripes according to the common practice of Royan Institute [27]; in addition, the decellularization process was started right after thawing the OTC. In order to decellularize OTCs, the ovarian tissue pieces were stored at -80˚C overnight and then placed and agitated in 0.5 M NaOH solution at room temperature [28]. Tissue stripes were then treated with a nuclease (RNase/DNase) supplemented solution (respectively 50 IU/mL, 1 IU/mL) in PBS for 24 h at 36 ˚C (Thermo Fisher, USA). They were then incubated on a rotating table at 37˚C for 24 h. To separate residual chemicals, phosphate buffered saline (PBS, Invitrogen, USA) washing was applied six times for 48 h. Next, some pieces of the DCTs were fixed for histological, DNA content and DAPI evaluation techniques and then the other pieces were cryopreserved in sterile H_2_O at − 80˚C for subsequent xenograft procedures (Fig. 1).

### Assessments of native and decellularized human ovarian cortical tissues

#### Morphological evaluations (H&E, DAPI)

**Fig. 2:**
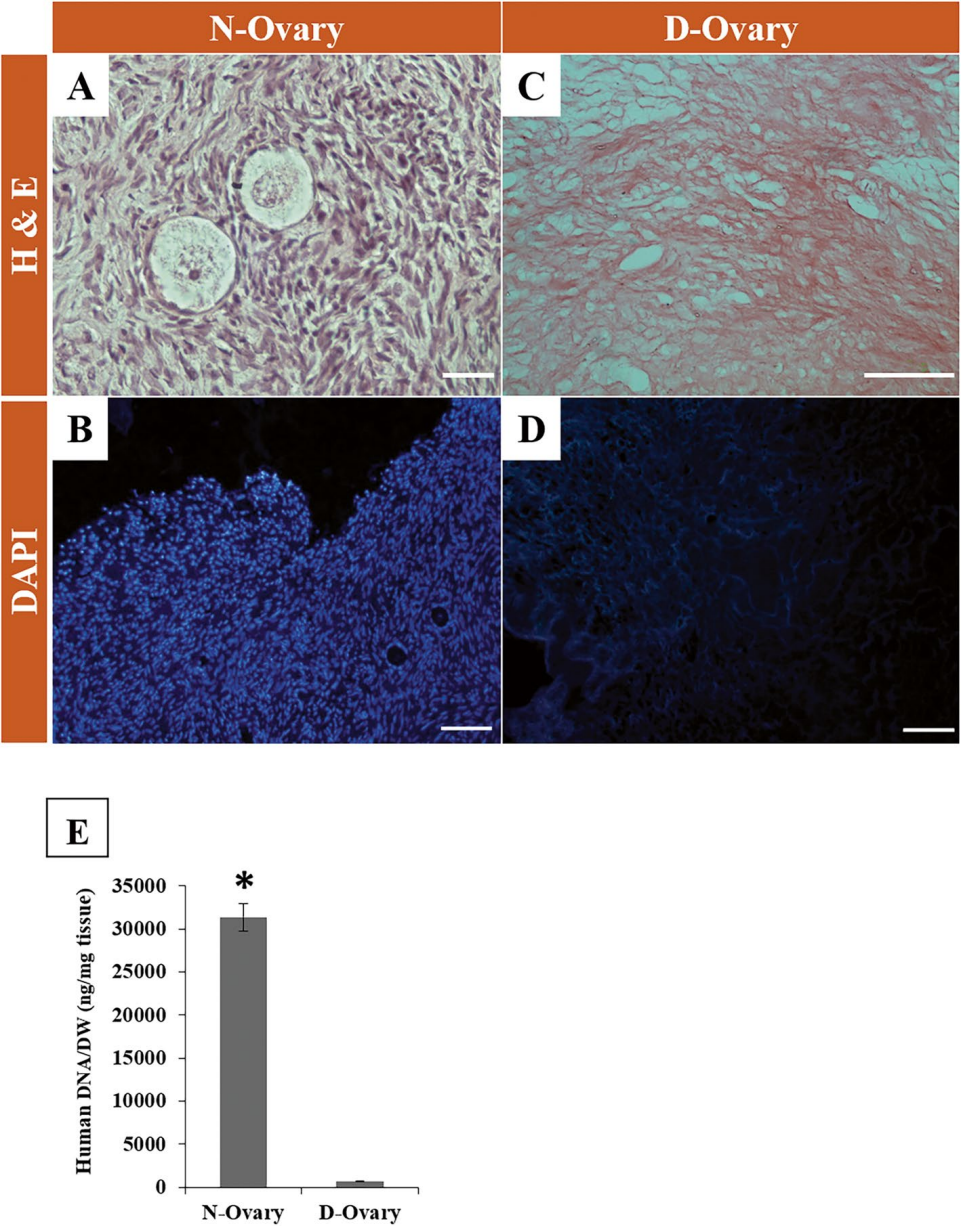
Setting up the decellularization protocol for human cortical ovarian tissues from Transsexual and Chemo-POF patients. **A** H&E and **B** DAPI staining on the native tissue (N-Ovary) and present both panels (A and B) from top to bottom. **C** H&E and **D** DAPI staining on the decellularized ovarian tissues (D-Ovary) after treatment with 0.5 M NaOH and subsequent DNase for 24 h. After 24 h of decellularization, nuclear remnants left, only the collagen fibers were present. **E** Relative DNA content in the native cortical (*N* = 9) tissues compared to decellularized corresponding tissues treated for 24 h. Asterisk: significant difference between the two groups (*P* < 0.05). Scale bars: A, B & D = 100 μm and C = 16 μm

The histology lab of Royan Institute hosted all the tissue processing steps for histological evaluations. Bouin's (4 h) and then 10% PBS/neutral buffered formalin solution (pH: 7.4) were used for fixing native and decellularized tissue fragments at room temperature (each one for 24 h). Fixed tissue pieces were washed in d.d.H_2_O (double distilled water) and dehydrated using a raised scaled alcohol series and then embedded in paraffin. Next, 6 μm thick sections were prepared from tissue samples using a microprocessor machine (Thermofisher, USA). H&E and 4,6-diamidino-2- phenylindole staining (DAPI, Sigma-Aldrich, D8417) were used for light microscopic (Nikon Eclipse 50i, Japan) analyses of the selected slides for morphological evaluations and for confirming the extent of decellularization, respectively (Fig. 2A-D).

#### Quantitative evaluation (DNA)

The total DNA remnants in the DCTs and untreated tissues from the same patients as baseline controls (native tissues) were quantified. The samples were completely homogenized and solubilized in 1 ml lysis buffer (50 mM tris–HCl, 50 mM EDTA, 1% SDS, 10 mM NaCl, pH: 8.0) and digested overnight in the presence of proteinase K, in a water bath at 65 ˚C, followed by a phenol/chloroform extraction. DNA was precipitated from the hydrous phase with 100% ethanol, after which, the extracts were washed with 70% ethanol. After dissolving the final pellet in RNase-free water, DNA content was determined in triplicates for each sample using a spectrophotometer (DU-730, Beckman Coulter, USA) at a wavelength of 280 nm. The amount of DNA in the DCTs and untreated tissues were averaged from a set of three independent runs, and the DNA content was then normalized to the initial wet weight of the tissue to obtain a correct estimation of tissue components. Additionally, the dry weights of the samples were measured in units of μg/mg (Fig. 2E).

#### MTT assay (Cytotoxic analysis of DCTs)

**Fig. 3:**
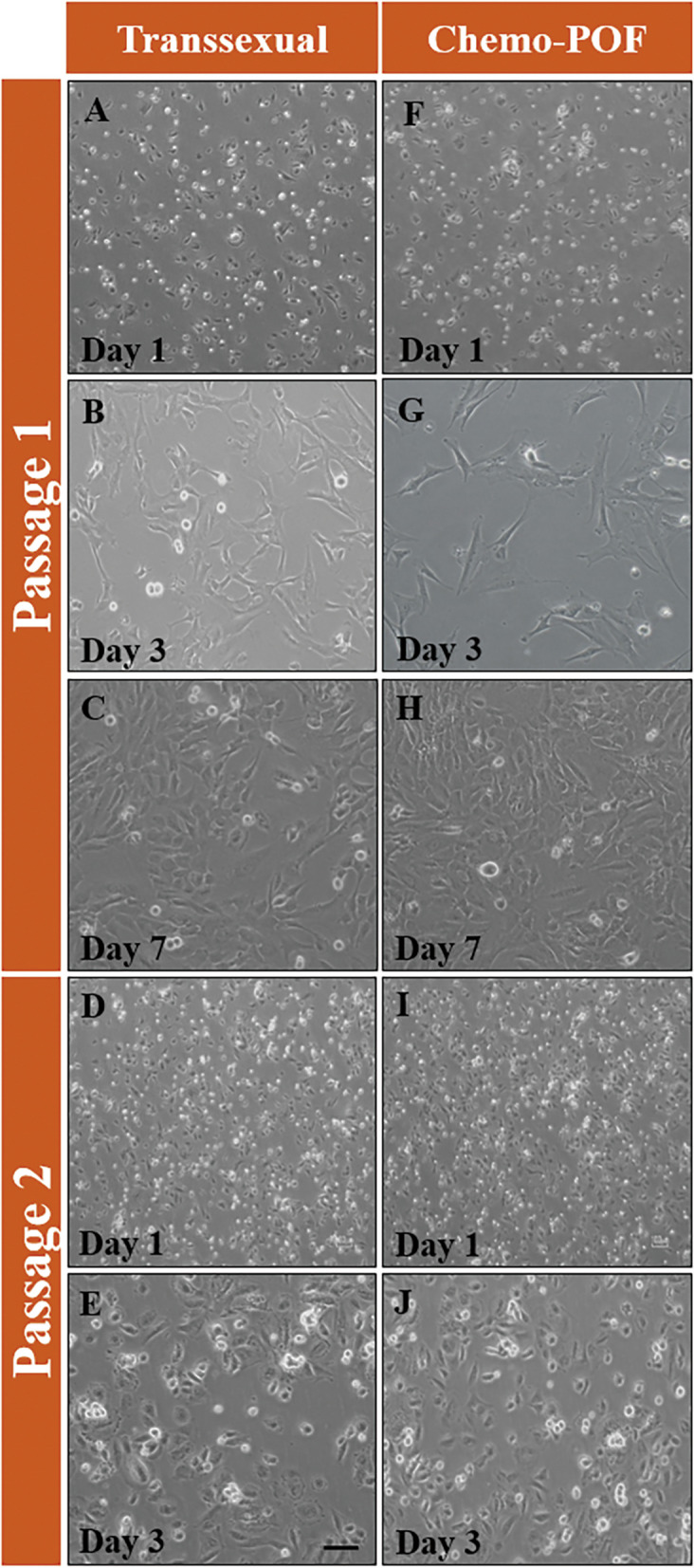
Morphology of HOCCs isolated from Transsexual and Chemo-POF ovaries. After ovarian digestion by collagenase I, dispersed ovarian cells were cultured. These figures illustrate the morphology cells cultured from transsexual (passage 1: **A-C**, passage 2: **D** and** E**) and Chemo-POF (passage 1: **F–H**, passage 2: **I** and **J**) ovaries at different days during culture. Scale bars: 200 μm

The cell viability and cytotoxicity of the scaffolds. were studied by 3-(4, 5-dimethylthiazolyl-2)-2, 5-diphenyltetrazolium bromide (MTT) assay (M5655; Sigma-Aldrich). The scaffolds (5 × 5 × 5 mm^3^) were first immersed in 70% ethanol for 30 min and then subjected to UV irradiation under a laminar flow. A total of 2 × 10^6^ HOCCs were plated in each well of 24-well plates. Then, the DCTs were loaded and co-cultured with human ovarian cortex cells suspension (HOCCs) and removed after 24, 48 and 72 h of culture. Next, the MTT solution (1 mg/mL of MTT in DMEM) was added and incubated for 3 h. Formazan crystals were then dissolved in 300 μl of dimethyl sulfoxide (DMSO; Sigma-Aldrich) for 15 min. The optical density (OD) was measured at a wavelength of 595 nm. Additionally, a conventional 2D monolayer and scaffold-free culture system on polystyrene 24-well dishes was used with the same cell density as the control (Fig. 3).

### Human ovarian cortex cells suspension procedure

Isolations of human ovarian cortical cells (HOCC_S_) from transsexual and Chemo-POF women (22–40 ages) were performed using the methods described in 2019 by Shahri PA et al. [29] with some modifications. This method was developed in our laboratory and was based on enzymatic dissociation. In brief, for each isolation, two pieces of frozen–thawed human ovarian cortex (5 × 5 × 5 mm^3^) were dissected with a bistoury into smaller parts in Hank's balanced salt solution with no calcium or magnesium (HBSS, BOSTER; Wuhan, China), containing collagenase IA (1.5 mg/ml; Sigma- Aldrich, UK), followed by incubation at 37 °C for 40 min under gentle agitation and pipetted every 15 min. The enzymatic digestion was completed using equal volumes of HBSS containing 1% penicillin–streptomycin (Gibco, USA) and 15% heat-inactivated fetal bovine serum (FBS, Gibco, USA). The cell suspension was filtered with a 70 and 40 μm cell strainer (Dutscher SAS, Brumath, France) to remove any residual connective tissue fibers. The cell suspension was centrifuged at 300 g for 5 min at 4˚C. The pellet was re-suspended in an appropriate volume of Dulbecco's Modified Eagle Medium (DMEM-F12, Gibco, cat#11,330,057). Finally, the cells were left in a T25 culture flask without a feeder layer. The medium in which the cells were cultured at 37˚C, consisted of DMEM/F-12, 15% FBS, 10 ng/ml EGF (epidermal growth factor; Invitrogen, MA, USA), 1 ng/ml bFGF (basic fibroblast growth factor; BD Biosciences, USA), 1 mM Glutamax (Gibco, USA), 1 mM non-essential amino acids (Gibco, USA), penicillin/streptomycin (Thermofisher, USA) 1 × concentrated and incubated in 97% humidity, 21% O2 and 5% CO2. Half of the medium was changed with fresh medium every 4 days, and the primary culture took 1 week for HOCC_S_ isolated from transsexual ovaries and 2 weeks for HOCC_S_ isolated from Chemo-POF ovaries (The reason for the longer primary culture duration in the case of Chemo-POF ovaries was due to the low number of cells isolated from these ovaries (. After achieving the 90% confluency, the primary culture was detached from the flask using 0.05% Trypsin/EDTA (Gibco) and subcultured in a ratio of 1:3 in each passage. HOCC_S_ after 2^nd^ passage were considered for the subsequent experiments and seeded into the DCTs (Fig. 3A-J).

### Assessments after isolating HOCCs from the human ovarian cortex (ICC, ALP, Real-time PCR)

#### Immunocytochemistry (ICC)

**Fig. 4:**
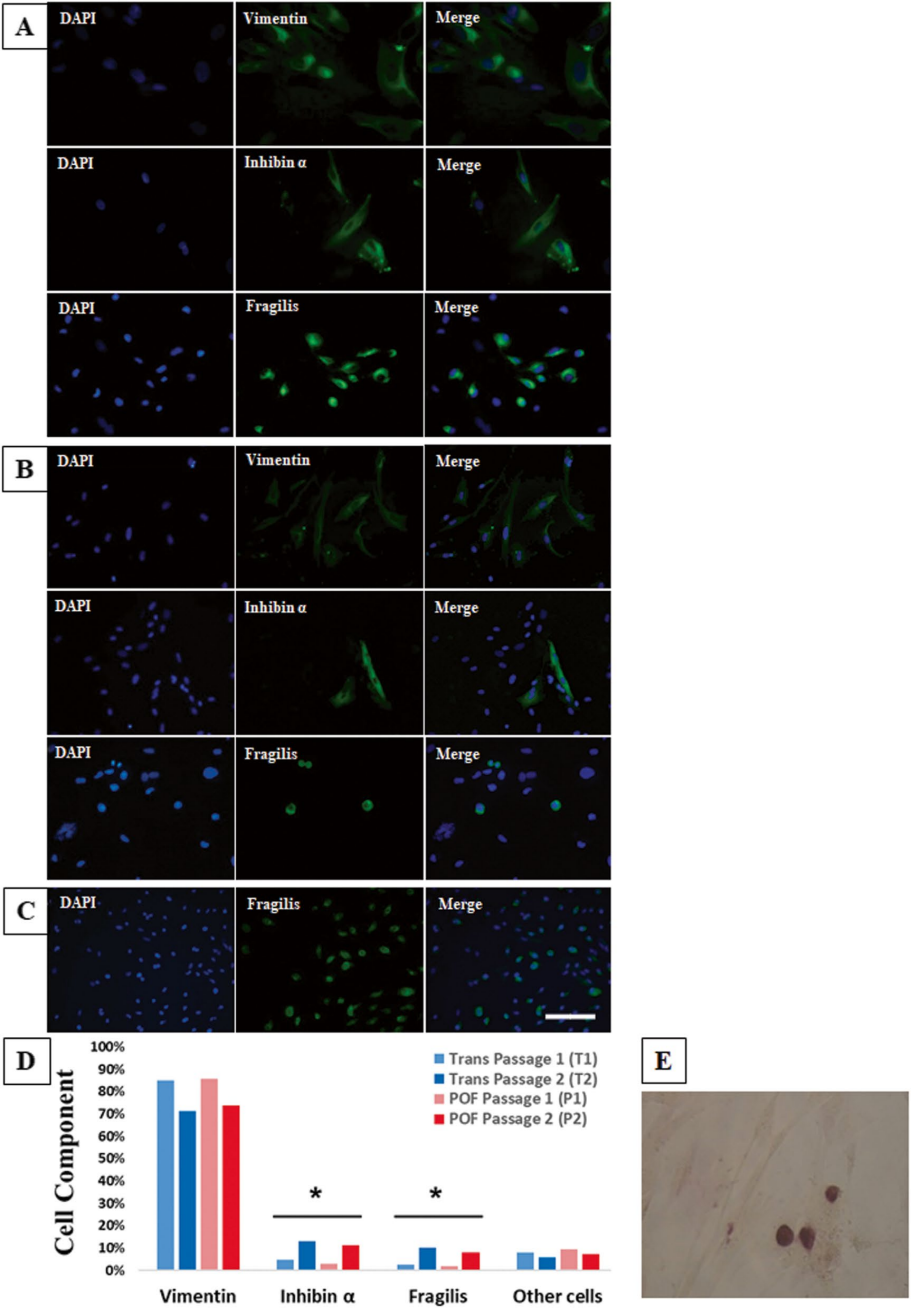
ICC analysis of the HOCCs isolated from transsexual (**A**) and Chemo-POF (**B**) ovary. Anti-human Vimentin ICC was used to localize and quantify human stromal cells in the HOCCs, Anti-human Inhibin α ICC was applied to localize and quantify human granulosa cells in the HOCCs and Anti-human Fragilis ICC was used to localize and quantify human OSCs in the HOCCs. **C** ICC analysis of OSCs isolated from transsexual ovary, after two passages, OSCs had increased in number. **D** Cell component comparison between Trans and Chemo-POF groups in passage 1 and passage 2. There are no significant differences in Vimentin positive cells between the groups in both passages 1 and 2. *: The Inhibin α and Fragilis positive cells have been significantly increased after passage 2 in both Trans and Chemo-POF groups (T1 with T2, T1 with P1, T2 with P2 and P1 with P2). **E** Alkaline phosphatase staining of OSCs. Scale bars: 50 μm. T1: Trans Passage 1; T2: Trans Passage 2, P1: POF Passage 1, P2: POF Passage 2

The HOCCs were cultured in 4-well plate until they reached 80–90% confluency, after which they were washed three times with PBS. The cells were fixed in 4% paraformaldehyde for 20 min at room temperature (RT). Next, the cells were washed with 0.05% PBS-Tween (PBST; 3 M Company, St. Paul, USA). The cells were incubated with 10% antigen retrieval for 20 min at 37˚C to expose the antigens, and then washed twice with PBST. Next, the samples were incubated with PBS containing 0.5% Triton X-100 for 15 min at RT to permeabilize the cells. Then, the cells were first blocked with a blocking solution for 1 h and incubated overnight at 4 °C with primary antibodies (Dissolved in 10% BSA and 10% PBS), anti-FRAGILIS (1:100; dilution, Abcam, USA), anti-Vimentin (1:100, dilution, Abcam, USA) and anti-Inhibin α (1:100; dilution, Abcam, USA). This information provides insight into the specific techniques used for evaluating the oogonial stem cells, stromal and granulosa cells proportion in ovarian cells for each group after isolation. The cells were first washed thrice with PBST and again incubated with FITC-conjugated rabbit anti-rabbit (1:500, dilution, Sigma-Aldrich, Germany), goat anti-mouse (1:500, dilution, Abcam, USA) or goat anti-rabbit (1:500, dilution, Abcam, USA) dissolved in 5% BSA in the dark for 1 h at 37C°. Then, the cells were washed with PBST, incubated for 1–2 min in DAPI followed by a double wash with PBST, and monitored using a fluorescence microscope (Olympus IX71). The cell numbers were calculated using Image J software to measure the number of positive cells. For negative controls, the slides were incubated with no primary antibodies (Fig. 4A-D).

#### Alkaline phosphatase staining (ALP) for characterizing OSCs

The purpose of conducting this staining was to identify and confirm the presence of ovarian stromal cells (OSC) within the HOCC samples. The HOCCs were cultured in a standard medium, and then they were fixed with 4% paraformaldehyde in PBS. Subsequently, the cells were stained using an alkaline phosphate (ALP) detection kit in accordance with the manufacturer's instructions (Merck Millipore, Middlesex, UK) (Fig. 4E).

#### Real-time PCR analysis of HOCCs (*DDX4, STELLA, FRAGILIS, Vimentin, FSH-R, KI67*)

To confirm the characteristics of HOCCs, real time PCR was carried out. Furthermore, total RNA was extracted from HOCC_S_ using Trizol reagent (Qiagen, USA), following the manufacturer's instruction. The extracted RNA sample quality was confirmed by electrophoresis, and its quantity was measured using a NanoDrop 2000 spectrophotometer (Thermo Scientific, Wilmington, DE).

To eliminate any trace DNA contamination, approximately 2 μg of RNA samples were treated with Dnase I RNase-Free Kit (EN0521; thermo scientific, Germany). Reverse transcription of mRNAs was then performed using the RevertAid-H Minus First Strand cDNA Synthesis Kit (Excell RT reverse transcription kit, RP1300, smo Bio, Krea) and random hexamer primer, according to the manufacturer’s instructions. Subsequently, the cDNA was stored at -80 ℃ until further use.

Then, specific primer pairs were used to study the expression of OSC markers (DDX4, STELLA, and FRAGILIS), a proliferation marker (KI67), a stromal cells marker (Vimentin), and a granulosa cells marker (FSH-R). The expression of GAPDH (qHsaCEP0041396) was used as a calibrator (housekeeping gene).

**Fig. 5:**
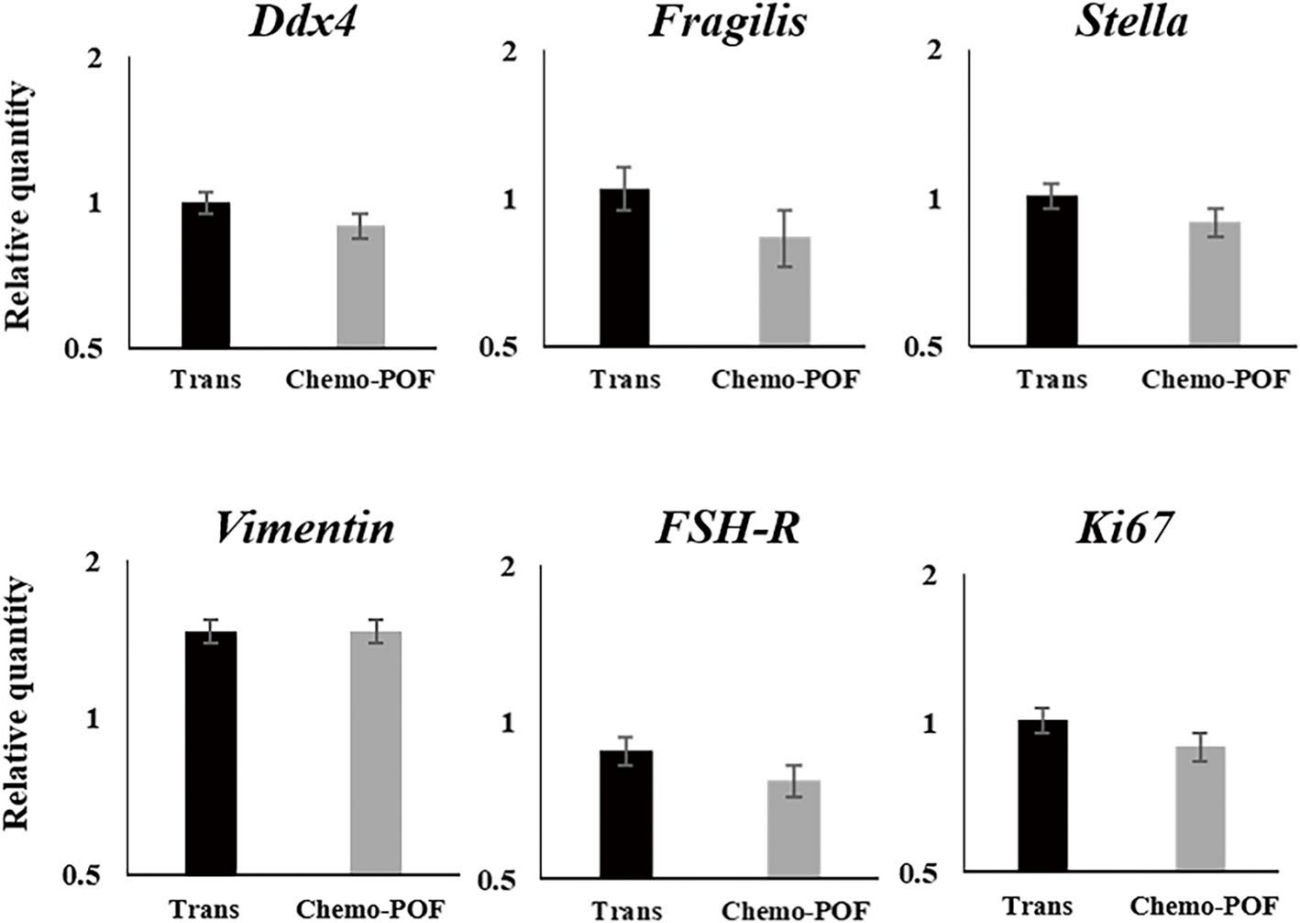
Analysis of gene expression in HOCCs (Human Ovarian Cortical Cells) before seeding into the scaffold: We conducted Real-Time PCR to assess the expression of specific markers in HOCCs, including markers for stromal cells (*Vimentin*), granulosa cells (*FSH*), germ cells (*Ddx4*, *Fragilis*, *Stella*) and cell proliferation (*Ki67*). The values are given as mean ± SEM

The PCR reactions were performed to amplify the target genes using specific primer pairs. The thermocycling parameters were set as follows: 15 min at 95 °C for enzyme activation, followed by 40 cycles of denaturation at 95 °C for 20 s, annealing at 60 °C for 60 s, and extension at 72 °C for 30 s using the Rotor-Gene Q instrument (Qiagen). Amplification was followed by melting curve analysis to verify PCR product specificity. Subsequently, the PCR product size was assessed by gel electrophoresis. All samples were run in duplicate, and the mean Ct-value of each duplicate was then used for further calculations. The reactions of no-template controls and studied samples were performed simultaneously. The comparative Ct method, 2^−ΔΔCt^, was used for relative gene expression analysis [30] (Fig. 5).

### Artificial ovary: seeding cells into the human ovarian DCTs

**Fig. 6:**
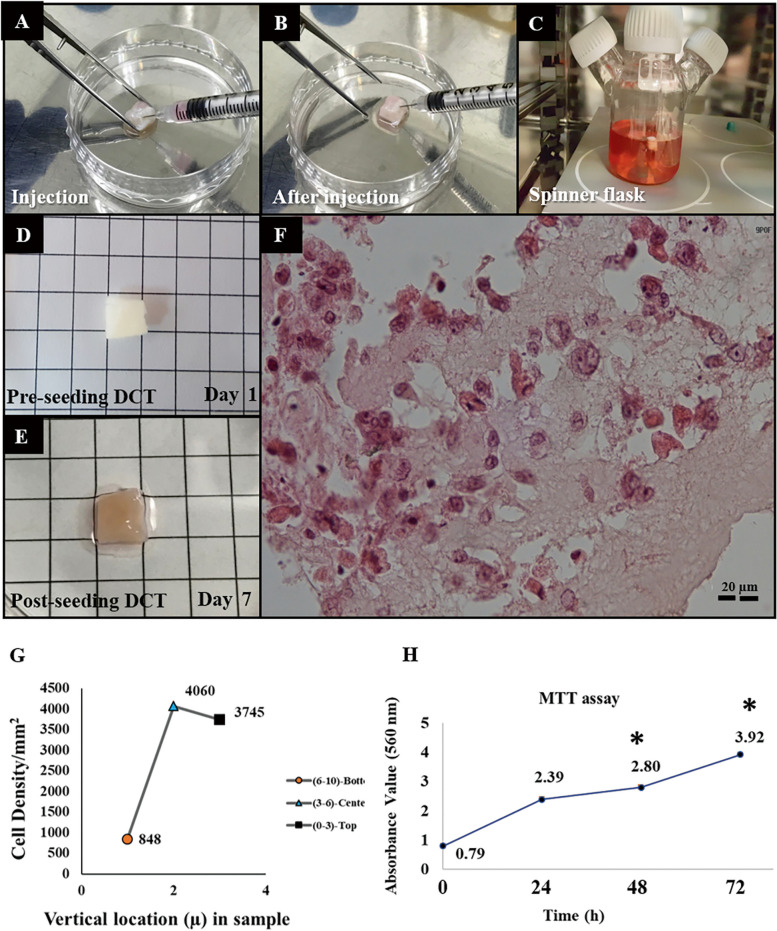
Seeding of DCT (Decellularized Cortical Tissue) with HOCCs (Human Ovarian Cortical Cells) in vitro. Seeding of DCT using two methods: **A** & **B** injection of HOCCs and **C** a spinner flask. **D** The morphology of pre-seeding DCT, **E** The morphology and **F** H&E staining of post-seeding DCT after 7 days of co-culture with HOCCs in spinner flask. **G** Stereology to count the cell density and zone division in DCT, including the exterior outer surfaces (top), the center of the section, and the bottom zone. **H** Cell viability using the MTT assay at 0, 24, 48 and 72 h after culturing the DCT. Asterisk: significant difference between the groups (*P* < 0.05). Values are given as mean ± SEM, Scale bar: 10 μm

Human ovarian bioscaffolds or artificial ovaries exhibit the potential to support human ovarian cortical cells both in vitro and in vivo. To sterilize the DCTs, they were soaked in 70% ethanol for two hours, washed with PBS, and exposed to 30 min of UV. Afterward, the DCTs were soaked in culture medium and kept in a 5% CO2 humidified atmosphere at 37 °C for one hour. After sterilization, the DCTs were supplemented with the cell suspension containing the HOCCs. The HOCCs were injected into the DCTs using an insulin syringe with a 30 G needle (DCTs + HOCCs = artificial ovary; AO) (Fig. 6A, B). The experimental groups were determined based on the source of HOCCs, which were isolated from both transsexual individuals (trans) and patients with chemotherapy-induced premature ovarian failure (Chemo-POF).

The seeded scaffolds were incubated for five hours in 21% O_2_, at 37 ˚C, 5% CO_2_, and 97% humidified in the Dulbecco’s modified eagle F12 (DMEM/F12) medium supplemented with 1% (5000 units/ml) penicillin/streptomycin (Gibco, USA), 15% FBS (Gibco, USA), 1% Glutamax (Gibco, USA), 10 ng/ml epidermal growth factor (EGF, Invitrogen), 1 ng/ml basic fibroblast growth factor (bFGF, Invitrogen) and 1% non-essential amino acid (Gibco, USA). Expansion of the HOCCs was conducted using a 100 ml spinner flask (Integra Biosciences™ 182,023, USA) with a stirring speed of 20 rpm to support cellular adhesion and distribution into the exterior and interior surfaces of the scaffolds. Fifteen scaffolds were placed in each spinner flask (2 × 10^6^ cells/scaffold) for each repetition. All artificial ovaries were cultured in 5% CO_2_ humidified atmosphere at 37˚C for one week and 50% of the medium was replaced every three days (Fig. 6C-E).

### Histological and stereological assessments of the artificial ovary

#### Morphological evaluations (H&E)

All histological tissue processing steps were carried out in the histology laboratory of the Royan Institute (Tehran, Iran). To prepare the artificial ovaries for histological analysis, each one was fixed initially in Bouin's solution for four hours, followed by a 24-h fixation in 10% neutral buffered formalin solution (pH: 7.4) at 25˚C. After fixation, the tissue was washed in distilled water, dehydrated in a graded series of alcohol, and then embedded in paraffin. Serial sections of tissues of 6 μm thickness were prepared using a microprocessor machine (Thermofisher, USA). For general examination of tissue morphology, selected slides were labeled on glass and stained with hematoxylin and eosin (H&E, Sigma-Aldrich). All images of AOs cross-sections were captured on an upright microscope (Olympus IX51, Japan) and cellular distribution and migration of seeded HOCCs were evaluated (Fig. 6F).

#### Stereological study (estimation of the cell density and number)

The numerical density of HOCCs was estimated using the optical dissector method. After culturing the AOs using rotational seeding, they were fixed in a fixative solution and embedded in a paraffin block. Twelve Sects. (20 μm) were randomly selected and mounted on slides, and H&E staining was performed to analyze each group. The sections were studied using a microscope with 100X magnification and a microcator system (ND 221 B, Heidenhain, Germany) connected to a computer was used to assess the Z axis. The nuclei of the HOCCs were sampled using an unbiased counting frame, ensuring that cells with their nucleus completely or partly inside the field, without touching the borders, were counted.

The numerical density “Nv” of HOCCs was estimated according to the following formula: Nv (cells) = [ΣQ/ (a/f × ΣP × h)], where “ΣQ” is the number of nuclei coming into the focus during scanning, "Σp" is total number of unbiased counting frames in all fields, “h” is the tissue thickness considered for counting and “a/f” is the frame area. Furthermore, the results were adjusted for the scaffold total volume (V) to obtain the total number of HOCCs in the AO (Fig. 6G, H).

### Ovariectomy and xenotransplantation of artificial ovaries to NMRI mice

**Fig. 7:**
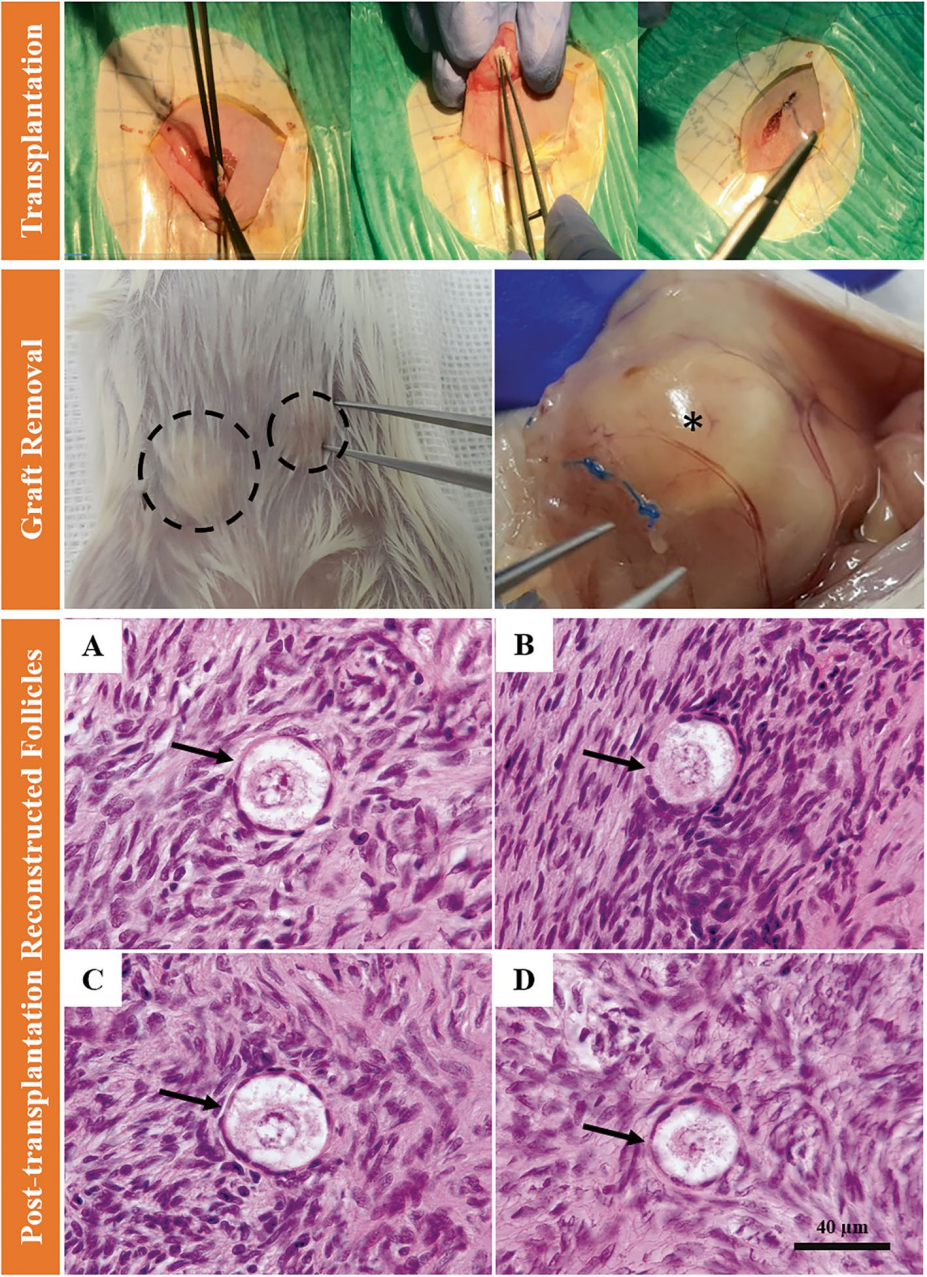
Human reconstructed ovarian follicles following 8 weeks xenotransplantation of HOCCs (Human Ovarian Cortical Cells) in ovarian decellularized scaffold. Bioengineered transplanted ovaries from over the skin (indicated by dashed lines) and after opening the skin (indicated by asterisk). Primordial and growing follicles in both the Trans (**A** and **B**) and the Chemo-POF group (**C** and **D**)

The non-immunodeficient mice were anesthetized with a combination of ketamine-xylazine (Alfasan, Netherland; 960λ-640λ/kg) injected intraperitoneally. The lateral abdominal wall was shaved and disinfected before being opened using two scissors. Xenotransplantation of AO (5 × 5 × 5 mm^3^) was immediately performed in the abdominal subserosal facial region of NMRI mice (*N* = 10) that had undergone ovariectomy through a ventral incision while under anesthesia. Next, the layers of abdominal wall were then closed using nonabsorbable suture (0.5 mm) (Teb Keyhan, Iran) for the skin and polyglycolic acid synthetic absorbable suture (0.6 mm) (Teb Keyhan, Iran) for the internal layers. After surgery, the mice were housed separately in cages with access to food and water in the Royan institute animal facility under standard conditions for two months (Fig. 7).

### Artificial ovary assessments after xenotransplantation

#### Histological and immunofluorescence assessments of AOs (H&E, IF)

**Fig. 8:**
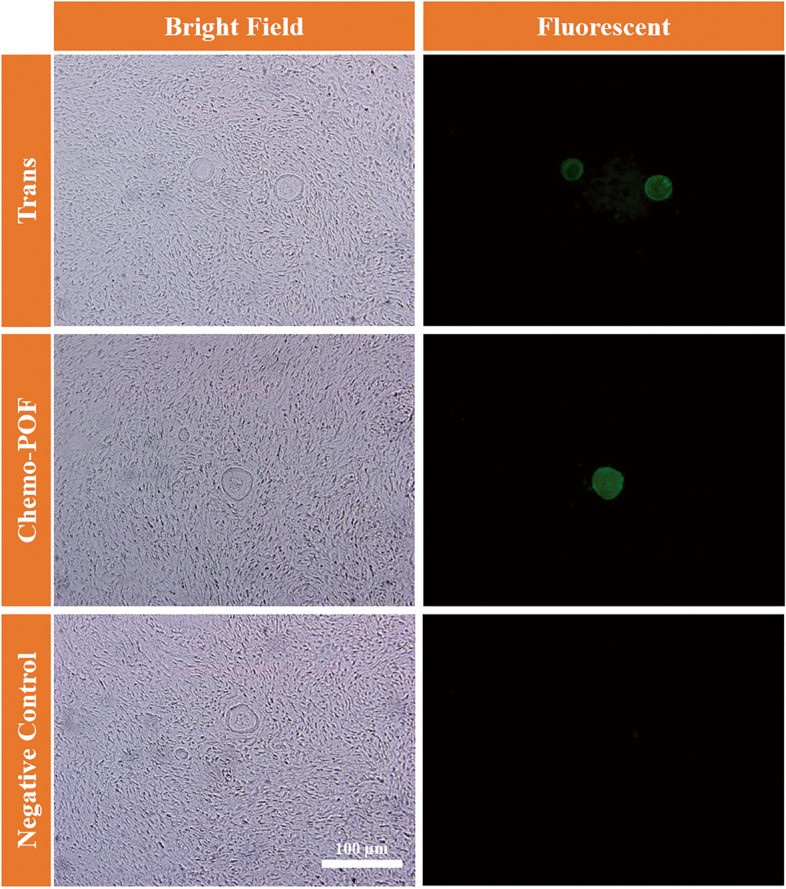
Immunofluorescence (IF) staining. IF was performed to detect the presence of GDF9 within reconstructed follicles located in the human DCT (Decellularized Cortical Tissue) after 8-weeks xenograft period

After two months, the transplanted animals were sacrificed via cervical dislocation. The grafts (AO: ECM scaffold + HOCCs) from both groups were detached from the body and fixed in formalin for H&E staining and immunofluorescence detection of GDF-9 positive cells. Then, H&E staining was carried out to assess the basic histomorphology and immunofluorescence studies were used to detect the specific marker (GDF-9) of reconstructed ovarian follicles in the transplanted grafts. H&E slides were observed under the light microscopy (Nikon eclipse 50i, Japan) (Fig. 7A-D), and IF using fluorescent microscopy (Olympus IX71) (Fig. 8).

#### Serum hormonal assay

**Fig. 9:**
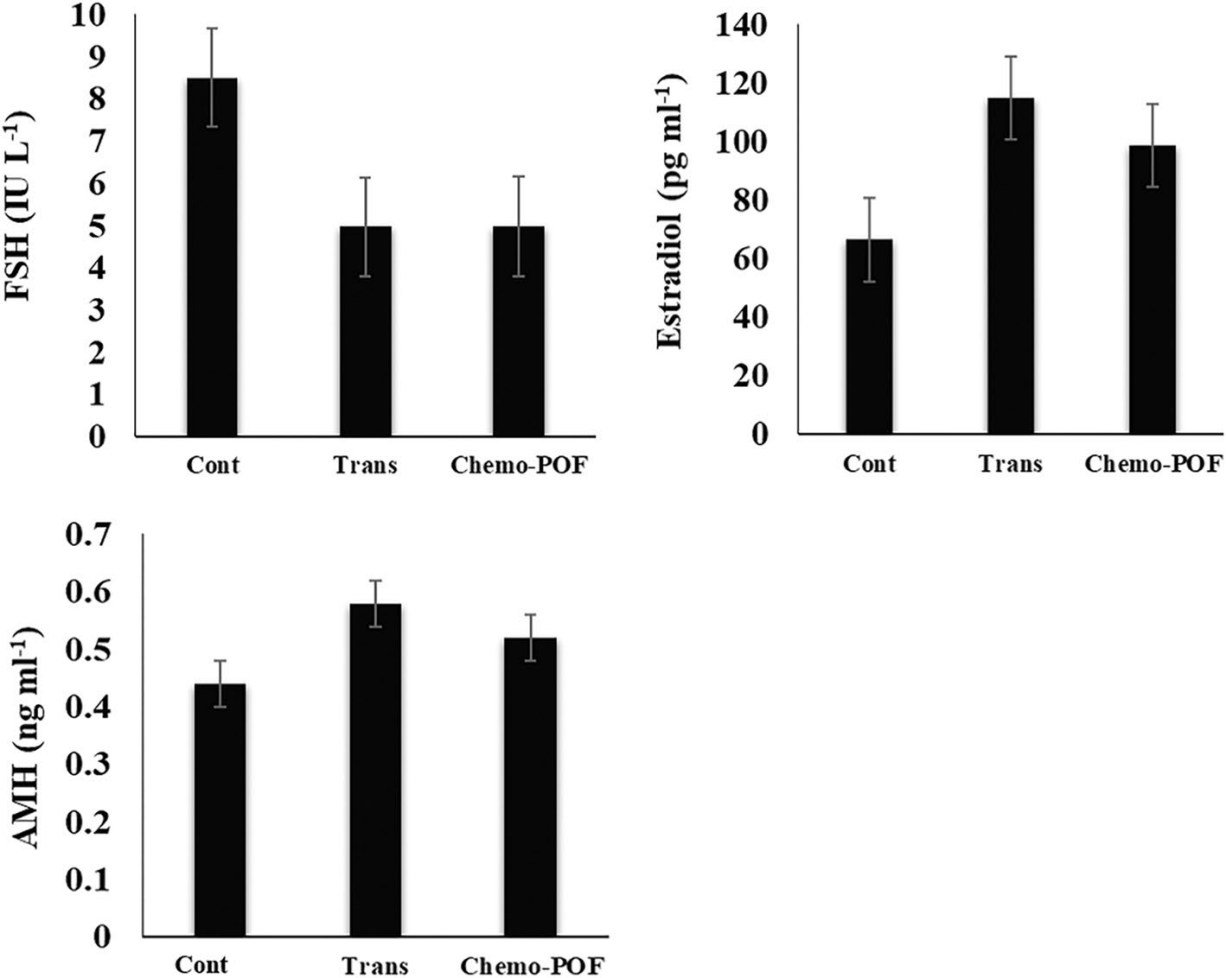
Hormonal assessment of artificial ovary, after 2 months of transplantation. The levels of estradiol, FSH (Follicle-Stimulating Hormone), and AMH (Anti-Müllerian Hormone) were determined by ELISA in all study groups. Additionally, we assessed hormonal changes in normal mice without any transplantation to provide a broader perspective on hormonal variations. Values are given as mean ± SEM

Two months after AO xenotransplantation, a hormonal assay was conducted on the serum of recipient mice. AMH level was assessed in the serum by the enzyme-linked immunosorbent assay (ELISA, CSB-E13156m, Houston, USA) method. The sensitivity of the AMH assay was 0.25 ng/ml with a reference range of 0.4 ng/ml to 14 ng/ml. The serum level of Follicle-Stimulating Hormone (FSH) was determined using a commercial ELISA kit (MBS2507988, Southern California, USA). The assay had a sensitivity was 0.94 ng/mL and a detection range of 1.56–100 ng/ml for FSH. Serum estradiol concentrations were determined using a commercial ELISA kit (#MBS261250, Southern California, USA) following the manufacturer's instruction, with a detection range was 15.6 to 1000 pg/ml. Bilateral ovariectomized mice without any graft was considered as a control group (Fig. 9).

#### Real-time PCR of transplanted AOs (*GDF9, ZP3, VEGF, CD34, KI67*)

**Fig. 10:**
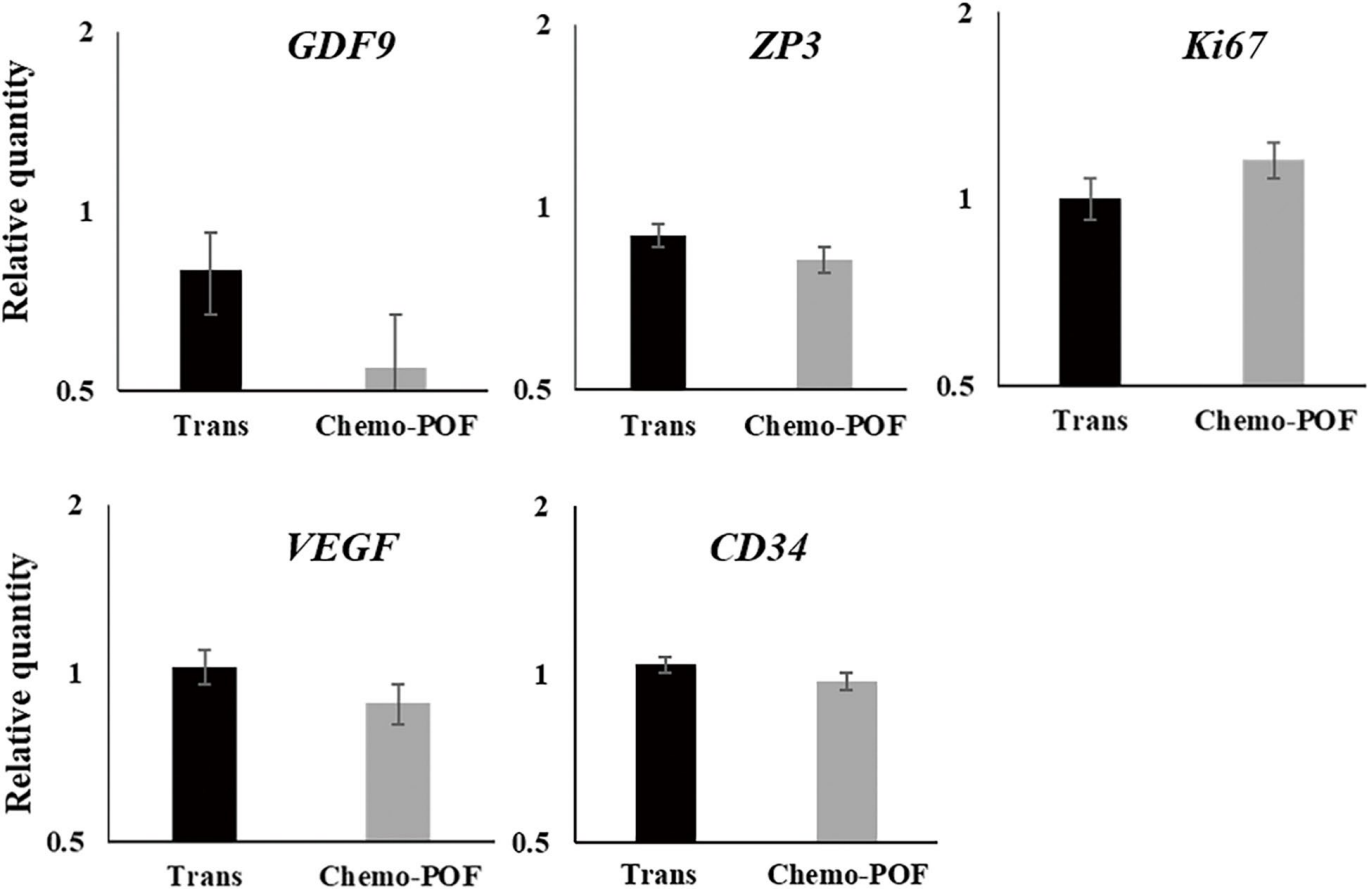
Analysis of gene expression in different cells. Gene expression was assessed in the artificial ovary two months after transplantation, specifically focusing on endothelial cells (VEGF, CD34), oocyte cells (GDF9, ZP3), and cell proliferation markers (Ki67) using Real-Time PCR (RT-PCR). Values are given as mean ± SEM

The total RNA was extracted from the graft, which consisted of the AO (ECM scaffold and HOCCs), following the instructions described above. PCR reactions were then performed using specific primers for various genes, including *GDF9* and *ZP3* (oocyte markers), *VEGF* and *CD34* (angiogenesis markers), *KI67* (a proliferation marker), and *GAPDH* (Fig. 10).

### Statistical analysis

Statistical analyses were performed using SPSS software, version 20.0 (SPSS Inc., Chicago, IL, USA). Group comparisons were made using the Pair T-test and one-way ANOVA. All data were precisely defined as the mean value plus-minus the standard error of the mean (mean ± SEM). A *p*-value of ≤ 0.05 was considered statistically significant in all analyses.

## Results

### Quality of ovarian decellularized cortical tissues (DCTs)

H&E staining revealed basophilic and eosinophilic staining in native ovarian tissues (Fig. 2A). In contrast, a large area of DCTs remained eosinophilic without any basophilic staining (Fig. 2C). Histological examination confirmed that all cells and their components had been eliminated from the DCTs and ECM had remained intact (Fig. 2C, D). The efficiency of NaOH treatment to remove nuclear residues was verified using the DAPI staining technique (Fig. 2B, D).

### Quantitative outcomes of decellularized ovarian cortex tissues

We quantitatively analyzed the DNA content of decellularized ovarian tissues. The results showed that approximately 95% of DNA was removed with this procedure in DCT samples compared to the native ones (*p* < 0.05) (Fig. 2E).

### In vitrocharacterization of HOCCs

Ovarian digestion was performed using collagenase enzyme and physical crushing in both Chemo-POF and transsexual women, resulting in the isolation of different types of cells including special granulosa cells, stromal cells, and OSCs. After 7–10 days of culture, the cells density reached over 80% confluency (Fig. 3A, B, C, F, G, and H). The cell suspensions were passaged twice to increase the number of HOCCs especially OSCs and cell colony formation showed a similar morphology to oogonial stem cells (Fig. 3D, E, I, J).

### Immunocytochemistry

The cells were identified in both transsexual (Fig. 4A) and Chemo-POF (Fig. 4B) groups with immunostaining, based on the expression of stromal, granulosa and oogonial stem cells markers such as Vimentin, Inhibin α and Fragilis, representatively. Based on the image J data of immunostaining, an increase in the number of OSCs was observed at the end of passage two (Fig. 4C). Also, cell component comparison between Trans and Chemo-POF groups in passage 1 and passage 2 showed no significant differences in Vimentin positive cells. Inhibin α and Fragilis positive cells have been significantly increased after passage 2 in both Trans and Chemo-POF groups (T1 with T2, T1 with P1, T2 with P2 and P1 with P2) (Fig. 4D). Although, the OSCs expressed alkaline phosphatase (ALP), the stromal and granulosa cells were negative for ALP expression (Fig. 4E).

### Real-time PCR analyses

The results showed that the stromal cells expressed *Vimentin*, granulosa cells expressed *FSH-R*, and the OSCs expressed germ cell genes like *DDx4*, *FRAGILIS*, and *STELLA*. Additionally, the proliferation index of HOCCs, as measured by the KI67 marker, was strongly associated with HOCCs growth (Fig. 5).

### 3D cell distribution in scaffolds cultured under rotational condition (spinner flask)

After incubating the ovarian samples with 0.5 M NaOH for over 24 h, followed by DNase treatment, the color of DCTs turned from red to white, indicating the elimination of cellular components from the tissue (Fig. 6D). Decellularized ovarian scaffolds were injected with HOCCs isolated from both transsexual and Chemo-POF women (Fig. 6A, B), followed by in vitro culture in a spinner flask for one week (Fig. 6C). The color of the assembled AOs changed from white to red due to cells seeding, proliferation and distribution within the scaffold (Fig. 6E). H&E staining was performed on serial sections of the AOs to assess the distribution of ovarian cells at different levels of DCTs (Fig. 6F).

After seven days of incubation, the cellular distribution and density were evaluated within the assembled AO produced by rotational seeding method. This method led to the distribution of ovarian cells throughout the DCT. The cellular distribution and density analysis showed that the number of HOCCs in the scaffold was 2,088,079 cells/mm^3^. The vertical distribution of cells in the cross section showed high density of cells in both the central (4060 cells/mm^2^) and superficial part (3745 cells/mm^2^) of the scaffold. The cell density diminished at the depth of the scaffold (848 cells/mm^2^) (Fig. 6G).

### Cytotoxic analysis of DCTs determination

The cytotoxicity assessment of residual chemicals in AO tissues was conducted after the decellularization process. Additionally, the viability and proliferation of HOCCs seeded into the scaffolds using rotational seeding were evaluated using an in vitro MTT assay. The MTT assay measured the optical density (OD) as a proxy for cell survival and confirmed that the decellularization process and rotational seeding methods did not affect the viability and proliferation of HOCCs (Fig. 6H).

### Evaluation of folliculogenesis in the artificial ovary

Similar to natural ovarian tissue, a functional transplantable AO would be capable of re-establishing both endocrine and reproductive functions following grafting. To evaluate this concept, the artificial ovaries were xenotransplanted into female NMRI mice (Fig. 7). Following two months of xenotransplantation, primordial and primary follicles were observed in the AO of both the Trans and Chemo-POF groups (Fig. 7).

### Immunofluorescence

The functionality and viability of the newly reconstructed ovarian follicles were confirmed by immunofluorescence analysis of growth differentiation GDF-9, which is associated with oocyte development, and by measuring the expression levels of GDF-9 (Fig. 8).

### Hormonal analysis

To evaluate endocrine activity, the levels of FSH, Estradiol and AMH hormones were assessed in 3 groups: ovariectomized mice without graft (Cont group) and with graft (Trans and Chemo-POF groups). The levels of Estradiol and AMH in Trans and Chemo-POF groups did not increase statistically compared to the Control. In addition, the serum FSH level in the groups with ovarian grafts showed a decrease non-significantly compared to the control group (Fig. 9).

### Real time PCR analyses

To confirm existing active follicles within the AO, total RNA was extracted from the artificial ovaries after two months of xenotransplantation and subjected to gene expression analysis. Real time PCR analysis showed full expression of follicle-related genes such as *GDF9* and *ZP3* in both the Trans and Chemo-POF groups. Although the relevant genes in the Trans group showed higher expression than in the Chemo-POF group, this the difference was not statistically significant between the two groups. Furthermore, both transplanted groups showed good expression of angiogenesis genes *VEGF* and *CD34*, indicating angiogenesis in the grafts. Additionally, the expression of *kI67*, a cell proliferation and survival factor confirmed cellular multiplication and health in the AO in both groups (Fig. 10).

## Discussion

In our study, we demonstrated that the reconstitution of human oogenesis and folliculogenesis using Human Ovarian Cortical Cells (HOCCs) within bioengineered artificial ovaries, incorporating a human ovarian Extracellular Matrix (ECM), could offer hope for fertility recovery in patients with compromised fertility due to cancer and its treatments.

Chemotherapy-induced changes in the tissue microenvironment that affect ovarian activity and functional links between microenvironmental proteins and cell differentiation. This can result in the depletion of ovarian reserve, which may ultimately lead to permanent ovarian failure [7–9]. The concept of the artificial ovary has recently opened new horizons in both suppressing cell metastasis and enhancing survival, growth rate, and microenvironment of ovarian cells and isolated follicles [12, 31]. An artificial ovary that combines biodegradable and biocompatible scaffold with cells can mimic the natural ovary and can be a promising approach to fertility recovery in patients with compromised fertility due to cancer and its treatments. Furthermore, the artificial ovary can restore gonadal hormone function without reintroducing malignant cells, which is a significant advantage over other fertility preservation methods such as ovarian tissue transplantation. In addition, the use of an artificial ovary can prevent delays in administering cancer therapy, since it avoids the need for ovarian stimulation and oocyte retrieval, which can delay cancer treatment by several weeks [32–36]. Our study demonstrates a functional link between bioengineered artificial ovaries using a human ovarian Extracellular Matrix (ECM) and Human Ovarian Cortical Cells (HOCCs), supporting the growth and differentiation of HOCCs into new oocytes. To create a human decellularized ovarian scaffold, we used a sodium hydroxide (NaOH)-treated protocol, which we then recellularized with HOCC_S_. We found that this approach resulted in the activation of oogenesis (follicle formation), causes neovascularization (*CD34*, *VEGF* expression*),* and restores gonadal hormone function (FSH, estradiol, AMH) in vivo.

A human decellularized ovarian scaffold was the initial step towards generating an artificial ovary that can support ovarian cells and mimics the natural environment [28]. The results of a previously established protocol by Eivazkhani et al. showed that NaOH is more effective than SDS for decellularizing human ovarian tissue, and leads to follicular reconstruction [28]. Our data is consistent with the results reported by Eivazkhani et al. and Hassanpour et al., which confirmed that the decellularization criteria were met, including the absence of visible nuclear material in tissue sections and a dsDNA content of < 50 ng dsDNA/mg dry ECM weight [18, 28]. Moreover, histological staining showed that decellularization and removal of the cell remnants were successful, and the 3D structure of the ECM and composition were well preserved after decellularization, aligning with findings from previous studies [18]. To assess the safety of NaOH detergent, cytotoxicity assays were performed by co-culturing HOCCs with DCTs for 24, 48, and 72 h. The results showed higher cellular viability and proliferation rates compared to the control group. The components of ECM can increase cellular viability and proliferation and promote cellular mitosis, proliferation, and recruitment by spreading cryptic peptides [28]. ECM releases many effective embedded bioactive molecules including growth factors such as VEGF. IGFs and FGFs, as well as anti-bacterial factors [37]. Furthermore, plays a crucial role in directing morphological organization and physiological functions by interacting with cell-surface receptors and binding to growth factors (GFs), which in turn elicit signal transduction and regulate gene transcription [38].

The second step toward generating an artificial ovary involved isolating suspensions of human ovarian cells (HOCCs). These cells play a fundamental role in the signal transduction and angiogenesis necessary for follicle formation. To support the proliferation and differentiation of OSCs into follicles, the microenvironment of the artificial ovary must be suitable for follicle growth and survival [22, 36]. Previous studies have demonstrated the differentiation capacity of bovine, mice, and human ovarian cells both in vitro and in vivo. However, none of these studies haven’t isolated cells from ovarian tissue of women with POF and cultured them on ECM scaffolds [18, 25, 28].

In the current study, isolated HOCCs from both transsexual and Chemo-POF human ovaries following a method established by Shahri PA et al. with a few modifications [29]. The HOCCs were characterized through the use of both immunofluorescence and real-time PCR evaluations. The HOCCs which contained granulosa, stromal, OSCs, and other ovarian cells, were cultured in vitro and showed an increase in cell number over time. This finding confirms the strong proliferation ability of OSCs under optimal culture conditions. The current study's results are consistent with earlier studies by Woods et al. (2013) and Silvestris et al. (2019) which demonstrated that the growth of OSCs in vitro can lead to the expansion of premeiotic germ cells and the spontaneous formation of immature oocytes [39, 40]. Moreover, Silvestris et al. (2018) demonstrated the successful production of oocyte-like cells through the differentiation of OSCs in vitro [41]. Further, in 2015, Xiong et al. isolated and cultured Female Germline Stem Cells (FGSCs) from ovarian tissue and showed that these cells grew in clusters during the early stages of subculture [42]. Moreover, OSCs have been shown to be capable of expanding, entering meiosis, undergoing de novo oogenesis, and supporting subsequent folliculogenesis. Additionally, stromal cells, which have been demonstrated to produce factors that positively influence folliculogenesis [43]. In this study, we cultured OSCs together with other ovarian tissue cells to investigate their interactions and potential effects on follicular development. In order to achieve the goal of maximizing differentiation and growth of germ cells, it is necessary to maintain them within an appropriate microenvironment.

The next step was to seed a suspension of human ovarian cells into the decellularized ovarian cortical tissues by direct injection and culturing in a spinner flask. This technique was used to evaluate the ability of the ovarian decellularized tissue to support the survival and growth of ovarian cells in vitro. Some researchers have posited that the decellularized ECM of the ovary can support and facilitate cell proliferation and adherence [14]. Our data is consistent with earlier studies that propose the auxiliary role of decellularized ovarian scaffolds from both human [18, 22, 23] and bovine [23] sources in supporting cellular physiological phenomena like vitality, growth, and differentiation.

After confirming the biological functions of the seeded HOCCs on scaffolds in vitro, DCT was seeded and then transplanted as an artificial ovary into ovariectomized NMRI female mice for a period of two months. The xenotransplantation of the AO into ovariectomized mice resulted in the reconstruction of the follicle, which was confirmed through the examination of histological sections after two months. The observations from the study demonstrate that the ECM is both biocompatible and regenerative. Furthermore, it stimulates the formation of follicles, which leads to the resumption of folliculogenesis in Chemo-POF patients who do not have follicles within their ovaries. Previous studies have shown that ECM scaffolds can mimic the microenvironment of the ovary and provide a suitable niche for cells and follicles to survive and function both in vitro and in vivo [14, 18, 25]. This study further demonstrates that not only can the ECM scaffolds support HOCCs growth, but it can also reinitiate gametogenesis.

An engineered ovary should be able to effectively mimic the functions of a natural ovary, including the induction of steroidogenesis and folliculogenesis [18, 28]. The results of the present study indicate that isolated ovarian cortical cells, free of follicles and derived from both trans- Chemo-POF women, can survive, proliferate, and differentiate within a human ovarian decellularized scaffold after xenotransplantation into mice. This novel strategy provides evidence for the possible simulation of ovarian function in vitro or in vivo, especially in patients with complete ovarian dysfunctions [44–46].

The result of real-time PCR showed neovascularization within xenotransplanted AO through the expression of *CD34* and *VEGF* markers. Additionally, the immunofluorescence technique revealed GDF-9 positive follicles, indicating the functionality and viability of these newly reconstructed ovarian follicles as well as neo-oogenesis. Moreover, hormonal assessment revealed declined levels of FSH in both the Trans and Chemo-POF groups after two months of xenotransplantation, indicating sexual recycling. The ECM scaffold can integrate and modify according to the needs for follicular formation [47, 48], and using immunodeficient mice instead of wild-type mice can provide a better understanding of this process.

## Conclusion

In conclusion, we discovered ovarian stem cells (OSCs) in the ovarian cortex of women with chemotherapy-induced premature ovarian failure (Chemo-POF). Furthermore, we successfully isolated and cultured these cells with human ovarian cortical cells (HOCCs) on an extracellular matrix (ECM) scaffold, creating an artificial ovary. After xenografting into mice, we observed that the OSCs were able to differentiate into new oocytes and reconstruct the follicular structure within the artificial ovary. Based on our findings, we believe that a bioengineered artificial ovary would represent a significant advancement in the field of reproductive tissue engineering.

## Data Availability

All data generated by this study are included in this manuscript.

## Abbreviations

POF: Premature Ovarian Failure
AO: Artificial Ovary
HOCCs: Human Ovarian Cortical Cells
DCT: Decellularized Cortical Tissue
ECM: Extracellular matrix
OSC: Oogonial stem cells
OTB: Ovarian Tissue Bank
NMRI: Naval medical research institute
PBS: Phosphate buffered saline
DAPI: 4,6-Diamidino-2- phenylindole staining
MTT: 3-(4, 5-Dimethylthiazolyl-2)-2, 5-diphenyltetrazolium bromide
DMSO: Dimethyl sulfoxide
OD: Optical density
FBS: Fetal bovine serum
DMEM: Dulbecco's Modified Eagle Medium
EGF: Epidermal growth factor
bFGF: Basic fibroblast growth factor
ICC: Immunocytochemistry
RT: Room temperature
PBST: PBS-Tween
ALP: Alkaline phosphatase
ELISA: Enzyme-linked immunosorbent assay
FSH: Follicle-Stimulating Hormone
NaOH: Sodium hydroxide
SLES: Sodium lauryl ester sulfate
GFs: Growth factors
FGSCs: Female Germline Stem Cells
