# Correction: Follicular reconstruction and neo-oogenesis in xenotransplantation of human ovarian isolated cells derived from chemotherapy-induced POF patients

**DOI:** 10.1186/s13036-024-00452-1

**Published:** 2024-10-07

**Authors:** Sara Khaleghi, Farideh Eivazkhani, Somayeh Tavana, Ashraf Moini, Marefat Ghaffari Novin, Petkov Stoyan, Hamid Nazarian, Rouhollah Fathi

**Affiliations:** 1 Department of Biology and Anatomical Sciences, School of Medicine, Shahid Beheshti University of Medical Sciences, Tehran, Iran; 2 Department of Embryology, Reproductive Biomedicine Research Center, Royan Institute for Reproductive Biomedicine, ACECR, Tehran, Iran; 3 Department of Endocrinology and Female Infertility, Royan Institute of Reproductive Biomedicine, ACECR, Tehran, Iran; 4 Breast Disease Research Center (BDRC), Tehran University of Medical Science, Tehran, Iran; 5 Platform Degenerative Diseases, German Primate Center, GmbH, Leibniz Institute for Primate Research, 37077 Göttingen, Germany; 6 German Center for Cardiovascular Research (DZHK), Partner Site Göttingen, 37077 Göttingen, Germany

**Correction: Journal of Biological Engineering (2023) 17:70**

10.1186/s13036-023-00384-2

The original article [1] unfortunately contained an error in the caption of Fig. 4: “IHC” should be corrected to “ICC”, in order for the caption to read:

Fig. 4 ICC analysis of the HOCCs isolated from transsexual (A) and Chemo-POF (B) ovary. Anti-human Vimentin ICC was used to localize and quantify human stromal cells in the HOCCs, Anti-human Inhibin α ICC was applied to localize and quantify human granulosa cells in the HOCCs and Anti-human Fragilis ICC was used to localize and quantify human OSCs in the HOCCs. C ICC analysis of OSCs isolated from transsexual ovary, after two passages, OSCs had increased in number. D Cell component comparison between Trans and Chemo-POF groups in passage 1 and passage 2. There are no significant differences in Vimentin positive cells between the groups in both passages 1 and 2. *: The Inhibin α and Fragilis positive cells have been significantly increased after passage 2 in both Trans and Chemo-POF groups (T1 with T2, T1 with P1, T2 with P2 and P1 with P2). E Alkaline phosphatase staining of OSCs. Scale bars: 50 µm. T1: Trans Passage 1; T2: Trans Passage 2, P1: POF Passage 1, P2: POF Passage 2.

The original article has been corrected.

